# Exenatide can inhibit calcification of human VSMCs through the NF-kappaB/RANKL signaling pathway

**DOI:** 10.1186/s12933-014-0153-4

**Published:** 2014-11-19

**Authors:** Jun-Kun Zhan, Pan Tan, Yan-Jiao Wang, Yi Wang, Jie-Yu He, Zhi-Yong Tang, Wu Huang, You-Shuo Liu

**Affiliations:** Department of Geriatrics, Institute of Aging and Geriatrics, The Second Xiangya Hospital, Central South University, 139 Middle Renmin Road, Changsha, Hunan 410011 P.R. China

**Keywords:** Arterial calcification, Vascular smooth muscle cells, Osteoblastic differentiation, Glucagon-like peptide-1, Diabetes

## Abstract

**Background:**

Arterial calcification is an important pathological change of diabetic vascular complication. Osteoblastic differentiation of vascular smooth muscle cells (VSMCs) plays an important cytopathologic role in arterial calcification. The glucagon-like peptide-1 receptor agonists (GLP-1RA), a novel type of antidiabetic drugs, exert cardioprotective effects through the GLP-1 receptor (GLP-1R). However, the question of whether or not GLP-1RA regulates osteoblastic differentiation and calcification of VSMCs has not been answered, and the associated molecular mechanisms have not been examined.

**Methods:**

Calcifying VSMCs (CVSMCs) were isolated from cultured human arterial smooth muscle cells through limiting dilution and cloning. The extent of matrix mineralization was measured by Alizarin Red S staining. Protein expression and phosphorylation were detected by Western blot. Gene expression of receptor activator of nuclear factor-κB ligand (RANKL) was silenced by small interference RNA (siRNA).

**Results:**

Exenatide, an agonist of GLP-1 receptor, attenuated β-glycerol phosphate (β-GP) induced osteoblastic differentiation and calcification of human CVSMCs in a dose- and time-dependent manner. RANKL siRNA also inhibited osteoblastic differentiation and calcification. Exenatide decreased the expression of RANKL in a dose-dependent manner. 1,25 vitD_3_ (an activator of RANKL) upregulated, whereas BAY11-7082 (an inhibitor of NF-κB) downregulated RANKL, alkaline phosphatase (ALP), osteocalcin (OC), and core binding factor α1 (Runx2) protein levels and reduced mineralization in human CVSMCs. Exenatide decreased p-NF-κB and increased p-AMPKα levels in human CVSMCs 48 h after treatment. Significant decrease in p-NF-κB (p-Ser^276^, p-Ser^536^) level was observed in cells treated with exenatide or exenatide + BAY11-7082.

**Conclusion:**

GLP-1RA exenatide can inhibit human VSMCs calcification through NF-κB/RANKL signaling.

## Introduction

Diabetes mellitus is a major risk factor for the development of cardiovascular disease [1]. Currently, treatment of diabetes and its cardiovascular complications with classic drugs is effective in less than 50% of patients [2]. Novel therapies are therefore required to manage diabetes mellitus and mitigate cardiovascular risks. GLP-1 is an incretin hormone that is rapidly released from intestinal L-cells after food intake. GLP-1 has insulinotropic effects, and it lowers blood glucose in a glucose-dependent manner [3]. Targeting of glucagon-like peptide-1 receptor (GLP-1R) has been developed to complement conventional treatment options for diabetes mellitus. This class of drugs is currently undergoing clinical trials for the treatment of type 2 diabetes.

Exenatide is a synthetic peptide sharing 53% sequence homology with GLP-1 and has been used as an agonist of mammalian GLP-1 receptor (GLP-1RA) for the treatment of type 2 diabetes [4,5]. In clinical trials, treatment with exenatide once a week assisted more patients in reaching the majority of ADA-recommended therapeutic goals than treatments with other drugs, such as sitagliptin, pioglitazone, or insulin glargine [6]. However, the residual adverse effect of sulphonylurea was also higher in patients who were treated with exenatide once a week [7]. Compared to conventional treatments, GLP-1RAs not only have an advantage in lowering blood sugar, but also have other benefits. For example, it has been reported that exenatide confers cardioprotection [4,5,8]. Arterial calcification is an important pathological change of diabetic vascular complication, which results in cardiovascular events and amputations [9]. However, the question of whether or not GLP-1RA regulates arterial calcification has not been answered, and the associated molecular mechanisms have not been examined.

Studies have suggested that arterial calcification is an active and tightly regulated process similar to bone formation [10,11]. The differentiation of vascular smooth muscle cell (VSMC) to an osteoblastic phenotype is a key component of the cytopathologic foundation of arterial calcification [12-15]. Calcifying VSMCs (CVSMCs) can spontaneously form calcification nodules and express osteoblast phenotype genes, such as ALP, OC, and Runx2 [16,17], which are often used together as a cell model in arterial calcification research [18]. Receptor activator of nuclear factor-κB ligand (RANKL) is a member of the tumor necrosis factor family and is important for bone remodeling [19]. Researchers have discoved that OPG-deficient mice develop severe osteoporosis and arterial calcification [20,21]. OPG inhibits nuclear kappa B (NF-κB) by binding RANKL. RANKL directly promotes calcification of VSMCs [22]. In addition, elevated serum OPG has been observed to be associated with vascular calcification in humans with T2DM [23]. The elevated serum OPG may interfere with RANK/RANKL interactions in the vascular wall. In cardiovascular diseases, RANKL is positively associated with circulating OPG [24].

In the present study, we investigated the effects of exenatide and RANKL in the osteoblastic differentiation and calcification of CVSMCs and examined the associated mechanisms.

## Materials and methods

### Cell culture and in vitro calcification

Human VSMCs were isolated from excess donor vasculature after kidney transplantations as previously described [25]. Briefly, the arterial tissues were removed and rinsed several times in Hanks’s balanced salt solution. The arterial tissues were then minced and digested in 5 ml of digestion solution (0.25 mg/ml soybean trypsin inhibitor, 0.125 mg/ml elastase, 2.0 mg/ml crystallized bovine albumin, 10 mg/ml collagenase I, and 15 mM HEPES) at 37°C for 45 min. The digested cells were filtered through a sterile 100-mM nylon mesh, centrifuged at 1,000 rpm for 10 min, and washed with Dulbecco’s modified Eagle’s medium (DMEM) containing high glucose concentration (4500 mg/L) and 10% FBS (Gibco-BRL). CVSMCs were isolated from cultured VSMCs when multicellular nodules spontaneously appeared. Briefly, cells were cloned by limiting dilution. Colonial cells were identified as CVSMCs by positive α-actin staining and by high ALP expression and formation of calcified nodules. This study was approved by the Ethics Review Board of Second Xiangya Hospital, Central South University. Written informed consent was obtained from patients.

### Mineralization assay

The extent of matrix mineralization in cultured CVSMCs was determined by Alizarin Red S staining. Briefly, cells were fixed with 4% formaldehyde for 10 min at room temperature and exposed to 2% Alizarin Red S for 5 min. Cells were then washed with PBS to remove excess dye. For quantitative analysis of Alizarin Red S staining, the dye was released from the cell matrix by incubating with cetyl-pyridinium chloride for 15 min. The amount of released dye was quantified by spectrophotometry at 540 nm. Results were normalized to the total amount of cellular proteins.

### Osteoblastic differentiation

Human CVSMCs were fixed in 4% paraformaldehyde, washed with Tris–Tween-20 buffer, and permeabilized for 20 min in buffer containing 0.1% triton-X. After blocking for 1 h, cells were incubated with primary antibodies overnight at 4°C, followed by secondary antibodies for 30 min. anti-ALP, anti-Runx2, and anti-OC primary antibodies and secondary antibodies were purchased from Santa Cruz Biotechnology (Santa Cruz, CA, USA).

To analyze the dose dependence of calcification on exenatide, CVSMCs were cultured with β-GP (10 mM) and 0, 100 pM, 1 nM, and 100 nM of exenatide in serum-free high glucose DMEM for 48 hrs. ALP activity was determined using the spectrophotometric measurement of p-nitrophenol level in the culture medium [26]. Osteocalcin level was measured using a radioimmunoassay kit (DiaSorin, Stillwater, MN, USA). The ALP activity and OC level were then normalized to the total amount of cellular protein.

### Silencing of RANKL by RNA interference

Three RANKL target sequences (S1: 5’-GTGCAGAAATGGCGAGAATAC-3’, S2: 5’-GGAUGGCUCAUGGUUAGAUTT-3’, S3: 5’-CGGAUCAGGAUGCAACAUATT-3’) were used to design and synthesize siRNA. A scrambled siRNA sequence was used as a control for siRNA transfection as previously described [27]. CVSMCs were plated onto 60-mm dishes and cultured in DMEM without antibiotics 24 hours before siRNA transformation. Cells were transfected with RANKL siRNA or control siRNA using Lipofectamine 2000 (Invitrogen, Grand Island, NY) according to the instructions of the manufacturer and continually cultured for 72 hours. Cells were then harvested for analyses of RANKL expression. Among the 3 siRNAs, siRNA derived from the S2 target sequence was the most effective in silencing RANKL expression and was used for further experiments in this study.

### Western blot

Protein concentration was measured using a BCA Protein Assay kit (Beyotime, Shanghai, China). 20-μg of total protein was loaded onto a 12% SDS-PAGE gel and transferred onto nitrocellulose membranes. After blocking with 5% non-fat milk for 1 h, membranes were incubated with primary antibody overnight at 4°C and subsequently incubated with HRP-labeled secondary antibody (1:2000 dilution) for 2 h at room temperature. Reactive proteins were detected using chemiluminescent reagents (Pierce, Rockford, IL, USA). To control for loading efficiency, the blots were stripped and reprobed with GAPDH (glyceraldehyde-3-phosphatedehydrogenase) antibody. Primary antibodies for RANKL, AMPKα, Phospho-AMPKα Thr^172^, ERK1/2, Phospho-ERK, JNK, Phospho-JNK, NF-κB p65, phospho-NF-κB (p-NF-κB Ser^276^, p-NF-κB Ser^529^ and p-NF-κB Ser^536^), and GAPDH were purchased from Santa Cruz Biotechnology (Santa Cruz, CA, USA).

### Statistical analysis

Data were presented as mean ± standard deviation (SD) and analyzed using Statistical Product and Service Solutions (SPSS, version 17.0). Differences between two groups were analyzed using Student’s t-test. P <0.05 was considered statistically significant.

## Results

### Effects of exenatide and RANKL-siRNA on osteoblastic differentiation and calcification of human CVSMCs

**Figure 1:**
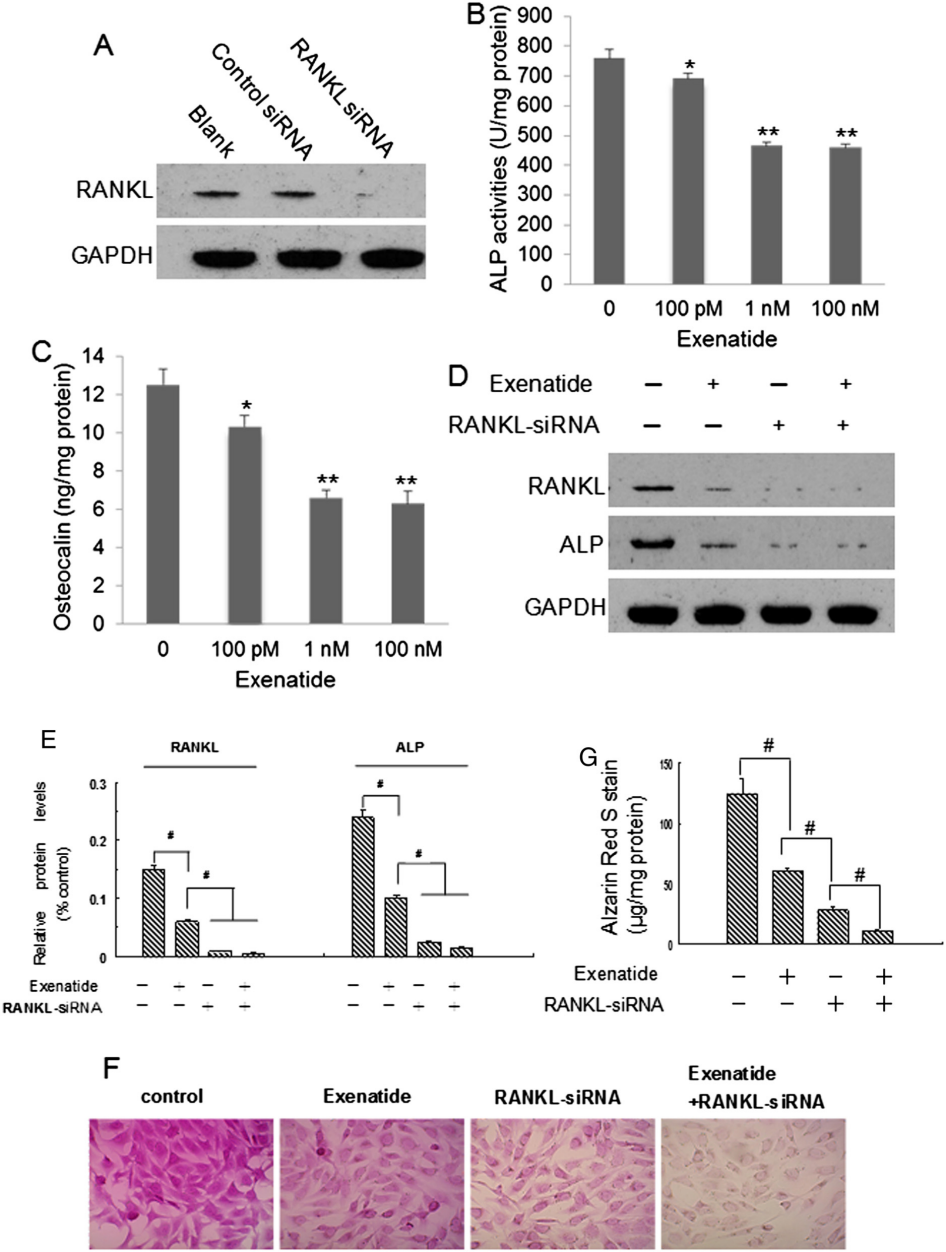
**Exenatide and RANKL-siRNA attenuate osteoblastic differentiation and calcification of human CVSMCs. A)** Representative Western blot of RANKL protein levels in human CVSMCs transfected with control siRNA (C-siRNA) or R-siRNA for 48 hours. **B**) Exenatide inhibited ALP activities in β-GP-treated VSMCs in a dose-dependent manner. VSMCs were treated for 48 hrs. N = 5, ^**^P <0.001 *vs.* control (0 concentration of exenatide). **C)** Exenatide inhibited OC levels in β-GP-treated VSMCs in a dose-dependent manner. VSMCs were treated for 48 hrs. N = 5, ^**^P <0.001 *vs.* 0 concentration of exenatide. **D)** Representative Western blot of ALP and RANKL protein expressions. Human CVSMCs were treated with or without exenatide (2 nM) and RANKL-siRNA (R-siRNA) for 48 h. GAPDH was used as the loading control. **E)** Semi-quantitative analysis of bands in Western blot at 48 hrs in each group. Bars represent mean ± SD. ^#^P < 0.001 between two indicated groups. N = 5. **F)** Representative Alizarin Red S staining. Human CVSMCs were treated with various agents for 15 days. Magnification × 200. **G)** Quantification of Alizarin red S staining. The dye was extracted and quantified as described in the Method section. Bars represent mean ± SD. ^#^P < 0.001 between two indicated groups. N = 5.

To determine the effect of GLP-1RA and RANKL on the osteoblastic differentiation and calcification of human CVSMCs, a GLP-1 receptor agonist (exenatide) and RANKL siRNA were used. Figure 1A shows that RNA interference efficiently knocked down RANKL gene expression in human CVSMCs. Treatment with siRNA-RANKL blocked the expression of RANKL protein in human CVSMCs. Further examination found that exenatide inhibited ALP activity (Figure 1B) and OC secretion (Figure 1C) in β-GP-treated VSMCs in a dose-dependent manner. Two nM of exenatide was selected for further studies. Two nM of exenatide downregulated ALP protein level in human CVSMCs compared to cells without exenatide treatment (Figure 1D and E). Moreover, treatment of human CVSMCs with exenatide + RANKL-siRNA or RANKL-siRNA alone downregulated ALP and RANKL protein levels (Figure 1D and E). Alizarin Red S staining showed weaker staining in CVSMCs treated with exenatide + RANKL-siRNA compared to control cells or cells treated with exenatide or RANKL-siRNA alone (Figure 1 F and G). These results indicated that exenatide treatment and RANKL siRNA attenuated the osteoblastic differentiation and calcification of human CVSMCs.

### Dose-effect relationship of the regulation of RANKL expression by exenatide in human CVSMCs and possible signaling pathways

**Figure 2:**
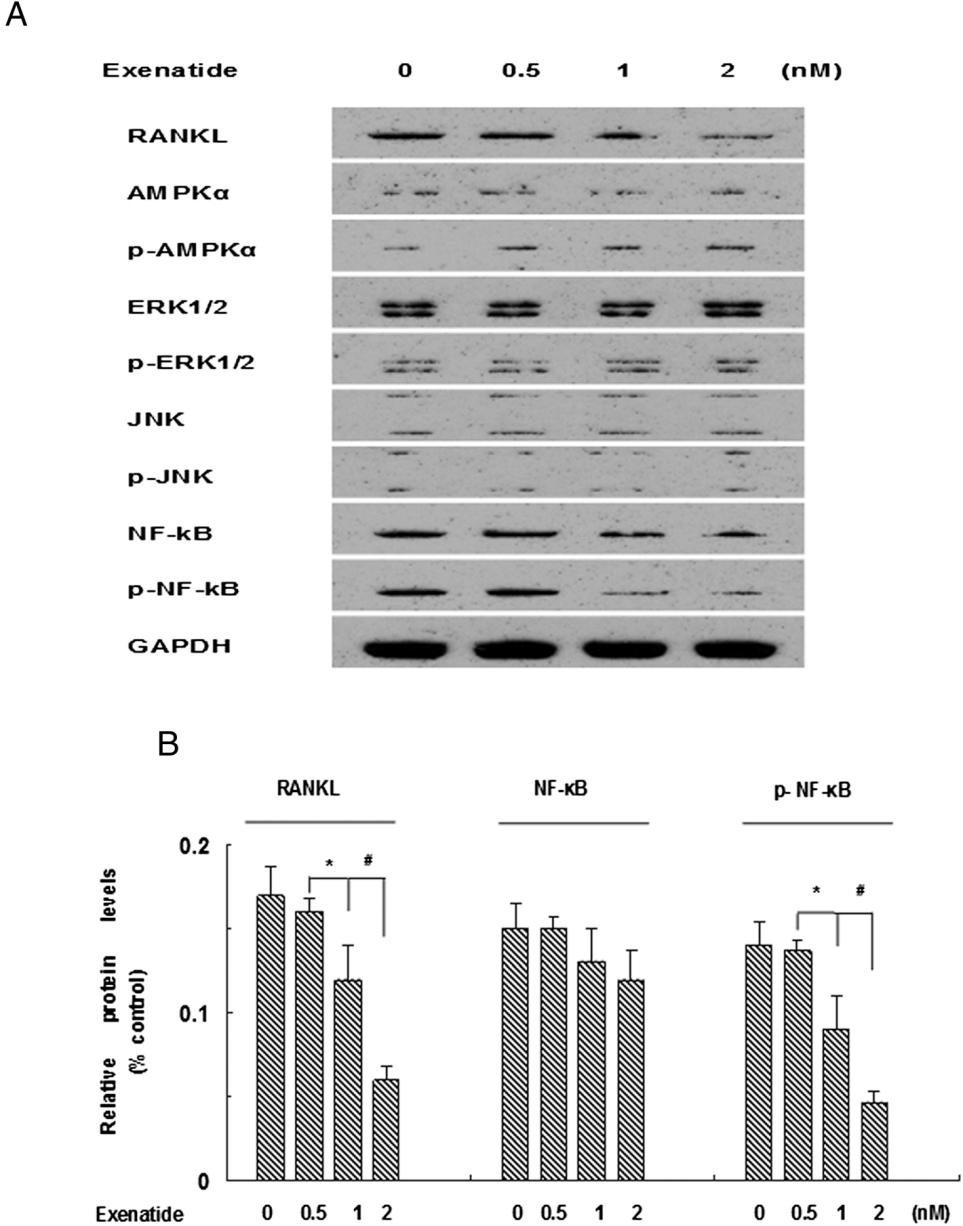
**Exenatide–inhibited osteoblastic differentiation and calcification of human CVSMCs requires NF-κB. A)** Human CVSMCs were treated with different concentrations of exenatide for 48 hrs. Representative Western blot of RANKL, AMPKα, p-AMPKα, ERK1/2, p- ERK1/2, JNK, p-JNK, NF-κB, p-NF-κB protein expression. **B)** Semi-quantitative analysis of bands in Western blot. GAPDH was used as the loading control. Bars represent mean ± SD. ^*^P < 0.01 and ^#^P < 0.001 between two indicated groups. N = 5.

Recent studies have demonstrated that several signaling pathways regulate RANKL expression in osteoblasts or VSMCs, such as AMPK, JNK, ERK1/2 and NF-κB signaling [11,28,29]. As shown in Figure 2A, exenatide decreased the expression of RANKL in a dose-dependent manner. Also, exenatide decreased p-NF-κB and increased p-AMPKα levels in human CVSMCs 48 h after treatment. In contrast, exenatide did not affect the protein level of AMPKα, ERK1/2, p-ERK1/2, JNK, p-JNK, or NF-κB (Figure 2).

### Effects of NF-κB inhibitor and RANKL activator on exenatide-inhibited osteoblastic differentiation and calcification of human CVSMCs

**Figure 3:**
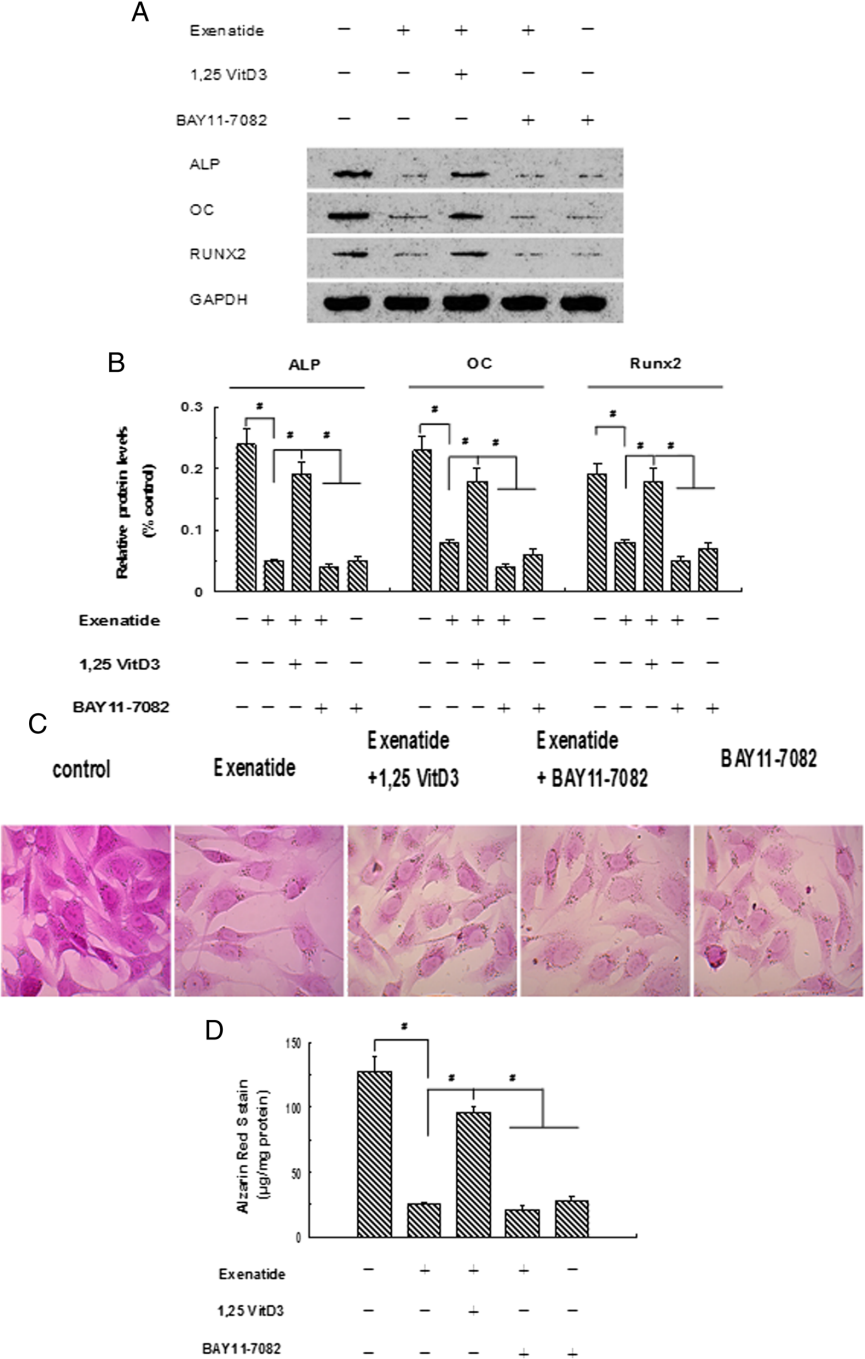
**Exenatide–inhibited osteoblastic differentiation and calcification of human CVSMCs can be regulated by NF-κB inhibitor and RANKL activator. A, B)** Human CVSMCs were treated with exenatide (2 nM), 1,25 VitD3 (10^-7^ M), and BAY11-7082 (10 μM) for 48 h. ALP, OC and Runx2 protein expressions were detected by Western blot. GAPDH was used as the loading control Bars represent mean ± SD. ^#^P < 0.001 between two indicated groups. N = 5. **C)** Human CVSMCs were treated with various agents for 15 days. The calcification was visualized by Alizarin Red S staining. Magnification × 200. **D)** Quantification of Alizarin red S staining. The dye was extracted and quantified as described in the Method section. Bars represent mean ± SD. ^#^P < 0.001 between two indicated groups. N = 5.

Human CVSMCs were treated with or without exenatide (2 nM), BAY11-7082 (an inhibitor of NF-κB, 10 μM), and 1,25-dihydroxyvitamin D3 (1,25vitD3) (an activator of RANKL, 10^-7^ M) [30] for 48 h. The expression of ALP, OC, and Runx2 proteins was detected by Western blot. Results showed that 2 nM of exenatide downregulated ALP, OC, and Runx2 protein levels in human CVSMCs compared to cells without exenatide treatment (Figure 3A and 3B). 1,25vitD_3_ upregulated ALP, OC, and Runx2 protein levels in human CVSMCs compared to cells treated with exenatide alone. Treatment of human CVSMCs with 2 nM of exenatide and 10 μM of BAY11-7082 more effectively downregulated ALP, OC, and Runx2 protein levels compared to cells treated with 2 nM of exenatide, 1,25 vitD_3_, or cells without treatment. Weaker Alizarin Red S staining was observed in CVSMCs cultured with exenatide alone than in cells cultured without exenatide. Stronger Alizarin Red S staining was observed in human CVSMCs cultured with exenatide and 1,25 vitD_3_ than in cells cultured with exenatide alone or exenatide + BAY11-7082 (Figure 3C and D). These results suggested that exenatide treatment may attenuate osteoblastic differentiation and calcification of human CVSMCs through NF-κB signaling. RANKL activation exhibits the opposite effect.

### Roles of RANKL and phosphorylation of NF-κB in exenatide-inhibited osteoblastic differentiation and calcification of human CVSMCs

**Figure 4:**
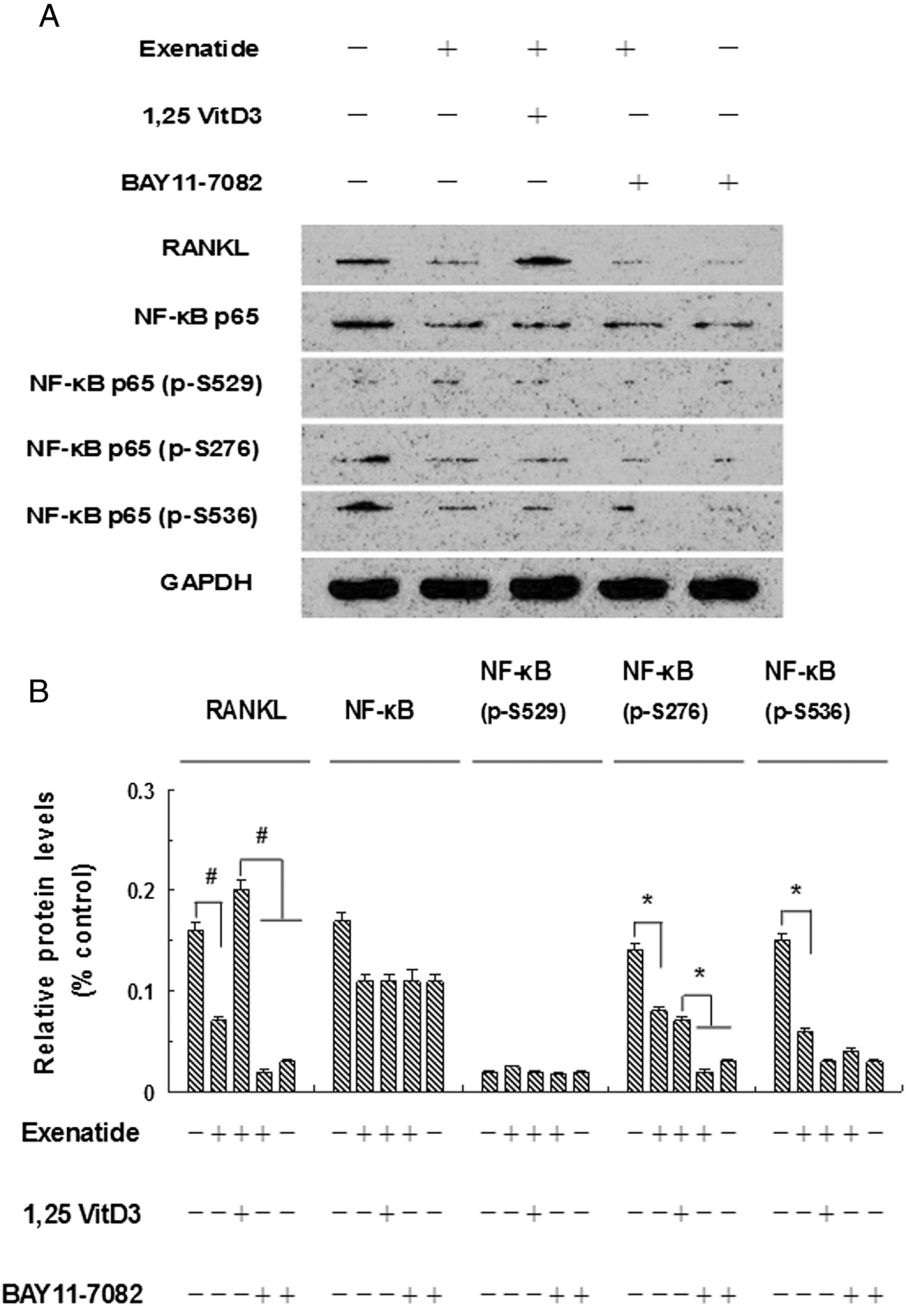
**The effect of exenatide in the phosphorylation of NF-κB and the expression of RANKL in human CVSMCs. A)** Human CVSMCs were treated with exenatide (2 nM),1,25VitD3(10^-7^ M) and BAY11-7082(10 μM) for 48 h. RANKL, NF-κB, p-NF-κB (S529, S276, S536) levels were detected by Western blot. GAPDH was used as the loading control. **B)** These results were reported at 48 h in each group. Bars represent mean ± SD. ^*^P < 0.01 and ^#^P < 0.001 between two indicated groups. N = 5.

We further investigated RANKL, NF-κB and p-NF-κB (p-Ser^529^, p-Ser^276^, p-Ser^536^) protein levels in human CVSMCs treated with or without exenatide (2 nM), BAY11-7082 (10 μM), and 1,25 vitD3 (10^-7^ M) (Figure 4A). The protein level of RANKL was significantly decreased in cells treated with exenatide alone compared to cells without exenatide treatment. Pretreatment of cells with the NF-κB inhibitor BAY11-7082 downregulated the protein level of RANKL. Conversely, pretreatment of cells with the RANKL activator 1,25 vitD_3_ upregulated the protein level of RANKL. These results indicated that RANKL is a downstream mediator for exenatide–reduced osteoblastic differentiation and calcification of CVSMCs. Western blot showed a significant decrease in p-NF-κB (p-Ser^276^, p-Ser^536^) level in cells treated with exenatide or exenatide + BAY11-7082 compared to cells in the no treatment group (Figure 4A and 4B). No changes in total NF-κB or NF-κB p65 (p-Ser^529^) levels were observed in human CVSMCs with different treatments. These results indicated that suppression of NF-κB phosphorylation, but not its expression, is necessary for exenatide-inhibited osteoblastic differentiation and calcification in human CVSMCs.

## Discussion

Type 2 diabetes mellitus is a progressive disease characterized by persistent insulin resistance and declining pancreatic β-cell function [31]. However, long-term glycemic control is hard to achieve because of deteriorating β-cell function. A multicentre randomized parallel-group trial suggested that exenatide was more effective in improving β-cell function compared to insulin and piogltazone; hence, early initiation of β-cell-protective therapy with exenatide may halt the decline in β-cell function in type 2 diabetes [32]. Indeed, two years after treatment initiation, patients on exenatide twice a day showed a significant reduction in body mass index and hemoglobin A1c compared with patients treated with a long-acting insulin analog [33]. In addition, even after only three months, exenatide therapy exhibited similar effects on microvascular endothelial function, inflammation markers, oxidative stress, and vascular activation as metformin in patients with obesity and pre-diabetes [34]. In this study, exenatide was able to inhibit human VSMCs calcification in cells cultured with high glucose concentration, suggesting that exenatide may exert its effect dependent on glucose concentration. Therefore, the detected alterations may play a role in the prevention of diabetes-induced vascular calcification.

Recent studies have suggested that GLP-1RA exhibits potential cardioprotective properties in addition to its role in lowering glucose level [35]. In animal models, exenatide has been demonstrated to be capable of protecting the heart against ischemia/reperfusion injury [4,36], reducing infarct size, improving cardiac function [4], and retarding atherosclerotic lesion formation [37]. Bao et al study demonstrated that sustained GLP-1 receptor activation plays an important role in providing cardioprotection in the setting of acute myocardial I/R injury [38]. However, the question of whether or not GLP-1RA protects the cardiovascular system by regulating VSMCs osteoblastic differentiation and calcification has not been answered, and the associated molecular mechanism is still unclear. Expression of ALP, OC, and Runx2 is a marker of osteoblastic differentiation, while matrix mineralization is a hallmark of the osteoblast phenotype [14,15]. In this study, we demonstrated that exenatide downregulated the expression of ALP, OC, and Runx2. In addition, exenatide inhibited mineralized nodule formation. We show for the first time that the GLP-1RA exenatide can inhibit osteoblastic differentiation and calcification of human CVSMCs.

Overexpression of RANKL has been found to stimulate VSMC mineralization [19-22,39]. However, most of the evidence for the direct role of RANKL signaling in arterial calcification is derived from animal studies. Little data is available on the role of RANKL signaling in relation to arterial calcification in human cells. The present study showed that exenatide inhibited RANKL expression in human CVSMCs through the NF-κB pathway and mitigated CVSMCs calcification. These findings suggest that exenatide inhibits the calcification of human CVSMCs via the NF-κB/RANKL signaling pathway.

The RANKL complex, consisting of the membrane-bound RANK, its ligand RANKL, and the decoy receptor OPG, is thought to be important for bone metabolism. Osteoblasts express OPG and RANKL, which regulate osteoclast differentiation through the activation of RANK [40]. The present study showed that exenatide could decrease the expression of RANKL and inhibit the expression of ALP, OC, and Runx2 in human CVSMCs. Furthermore, inhibition of RANKL expression by siRNA abrogated the role of exenatide in ALP expression in human CVSMCs. These findings suggest that RANKL is a key mediator in exenatide-induced inhibition of osteoblastic differentiation of human CVSMCs.

The NF-κB, MAPK, ERK, and NFAT signaling pathways have been demonstrated to regulate RANKL expression in osteoblast cells [29,41] and VSMCs [11,28]. In this study, exenatide inhibited the activation of NF-κB in human CVSMCs. Pretreatment of cells with NF-κB inhibitor inhibited the expression of RANKL in human CVSMCs. The NF-κB inhibitor BAY11-7082 abolished the effect of RANKL activator (1,25 vitD_3_) - promoted RANKL expression. In contrast, no changes in AMPKα, ERK1/2, p-ERK1/2, JNK, and p-JNK protein level have been observed in this study.

The NF-κB family consists of five members, RelA (p65), RelB, NF-κB1 (p50), NF-κB2 (p52), and c-Rel. p65 heterodimer is the predominant form in most cell types. The activation of NF-κB p65 is mediated by phosphorylation at Ser^529^, Ser^536^ and Ser^276^ residues [42]. However, the different functions of phosphorylation at these residues in arterial calcification have not been identified. In this study, we revealed that blocking NF-κB p65 phosphorylation at Ser^536^ and Ser^276^, but not at Ser^529^, abolishes NF-κB p65 activity. These findings provide direct evidence that phosphorylation of NF-κB p65 at Ser^536^ and Ser^276^ is required for β-GP-induced NF-κB activation in the osteoblastic differentiation of human CVSMCs.

## Conclusion

In conclusion, GLP-1RA exenatide can inhibit osteoblastic differentiation and calcification of human CVSMCs through the NF-κB/RANKL signaling pathway. This finding may represent a new therapeutic potential in arterial calcification for GLP-1RA, which is currently undergoing clinical trials for the treatment of type 2 diabetes.

## Abbreviations

β-GP: β-glycerol phosphate
ALP: Alkaline phosphatase
OC: Osteocalcin
Runx2: Core binding factor α1
VSMCs: Vascular smooth muscle cells
CVSMCs: Calcifying vascular smooth muscle cells
NF-κB: Nuclear Factor κB
RANKL: Receptor activator of nuclear factor-κB ligand
GLP-1RA: Glucagon-like peptide-1 receptor agonists
GLP-1R: Glucagon-like peptide-1 receptor
CVSMCs: Calcifying vascular smooth muscle cells
SPSS: Statistical package for the social science
siRNA: Small interference RNA
MAPK: Mitogen-activated protein kinase
AMPK: AMP-activated protein kinase
JNK: c-Jun N-terminal kinase
OPG: Osteoprotegerin
ERK: Extracellular signal-regulated kinase
RANK: Nuclear factor κB receptor activator
NEAT: Nuclear factor of activated T-cells
